# Turmeric and Its Major Compound Curcumin on Health: Bioactive Effects and Safety Profiles for Food, Pharmaceutical, Biotechnological and Medicinal Applications

**DOI:** 10.3389/fphar.2020.01021

**Published:** 2020-09-15

**Authors:** Javad Sharifi-Rad, Youssef El Rayess, Alain Abi Rizk, Carmen Sadaka, Raviella Zgheib, Wissam Zam, Simona Sestito, Simona Rapposelli, Katarzyna Neffe-Skocińska, Dorota Zielińska, Bahare Salehi, William N. Setzer, Noura S. Dosoky, Yasaman Taheri, Marc El Beyrouthy, Miquel Martorell, Elise Adrian Ostrander, Hafiz Ansar Rasul Suleria, William C. Cho, Alfred Maroyi, Natália Martins

**Affiliations:** ^1^ Zabol Medicinal Plants Research Center, Zabol University of Medical Sciences, Zabol, Iran; ^2^ Department of Agriculture and Food Engineering, School of Engineering, Holy Spirit University of Kasli, Jounieh, Lebanon; ^3^ Faculty of Medicine, American University of Beirut, Beirut, Lebanon; ^4^ Institut Jean-Pierre Bourgin, AgroParisTech, INRA, Université Paris-Saclay, Versailles, France; ^5^ Department of Analytical and Food Chemistry, Faculty of Pharmacy, Al-Andalus University for Medical Sciences, Tartous, Syria; ^6^ Department of Pharmacy, University of Pisa, Pisa, Italy; ^7^ Interdepartmental Research Centre for Biology and Pathology of Aging, University of Pisa, Pisa, Italy; ^8^ Institute of Human Nutrition Sciences, Warsaw University of Life Sciences, Warszawa, Poland; ^9^ Noncommunicable Diseases Research Center, Bam University of Medical Sciences, Bam, Iran; ^10^ Student Research Committee, School of Medicine, Bam University of Medical Sciences, Bam, Iran; ^11^ Aromatic Plant Research Center, Lehi, UT, United States; ^12^ Department of Chemistry, University of Alabama in Huntsville, Huntsville, AL, United States; ^13^ Phytochemistry Research Center, Shahid Beheshti University of Medical Sciences, Tehran, Iran; ^14^ Department of Pharmacology and Toxicology, School of Pharmacy, Shahid Beheshti University of Medical Sciences, Tehran, Iran; ^15^ Department of Nutrition and Dietetics, Faculty of Pharmacy, University of Concepcion, Concepcion, Chile; ^16^ Unidad de Desarrollo Tecnológico, UDT, Universidad de Concepción, Concepción, Chile; ^17^ Medical Illustration, Kendall College of Art and Design, Ferris State University, Grand Rapids, MI, United States; ^18^ Department of Agriculture and Food Systems, The University of Melbourne, Melbourne, VIC, Australia; ^19^ Department of Clinical Oncology, Queen Elizabeth Hospital, Kowloon, Hong Kong; ^20^ Department of Botany, University of Fort Hare, Alice, South Africa; ^21^ Faculty of Medicine, University of Porto, Porto, Portugal; ^22^ Institute for Research and Innovation in Health (i3S), University of Porto, Porto, Portugal

**Keywords:** *Curcuma longa* L., curcuma, turmeric, spice, curcuminoids, pharmacological effects, biotechnological applications

## Abstract

Curcumin, a yellow polyphenolic pigment from the *Curcuma longa* L. (turmeric) rhizome, has been used for centuries for culinary and food coloring purposes, and as an ingredient for various medicinal preparations, widely used in Ayurveda and Chinese medicine. In recent decades, their biological activities have been extensively studied. Thus, this review aims to offer an in-depth discussion of curcumin applications for food and biotechnological industries, and on health promotion and disease prevention, with particular emphasis on its antioxidant, anti-inflammatory, neuroprotective, anticancer, hepatoprotective, and cardioprotective effects. Bioavailability, bioefficacy and safety features, side effects, and quality parameters of curcumin are also addressed. Finally, curcumin’s multidimensional applications, food attractiveness optimization, agro-industrial procedures to offset its instability and low bioavailability, health concerns, and upcoming strategies for clinical application are also covered.

## Introduction

### A Brief Overview of *Curcuma* Species

The *Curcuma* genus has a long history of medicinal applications (Akarchariya et al., 2017; Dosoky and Setzer, 2018), being composed of approximately 120 species. Among the *Curcuma* species, *Curcuma longa L*. (Curcuma; Turmeric) is the most widely recognized; a cultivated plant, grown in a warm climate, in many regions of the world (Wu, 2015). However, the taxonomic identity of this genus is very difficult because of its extremely short period of flowering and herbarium preparation due to the flashiness of tubers, rhizomes, and inflorescence (Jadhao and Bhuktar, 2018). Rhizomes are the most commonly used plant part (Lakshmi et al., 2011), composed of a wide variety of compounds, including the bioactive non-volatile curcuminoids (curcumin, dimethoxy-, and bisdemethoxy-curcumin) and the compounds present in volatile oil (mono and sesquiterpenoids) (Itokawa et al., 2008; Lobo et al., 2009).

A multitude of beneficial pharmacological properties have been granted to the *Curcuma* species, including antiproliferative, anti-inflammatory, anticancer, antidiabetic, hypocholesterolemic, anti-thrombotic, antihepatotoxic, anti-diarrheal, carminative, diuretic, antirheumatic, hypotensive, antimicrobial, antiviral, antioxidant, larvicidal, insecticidal, antivenomous, and antityrosinase effects, among others (Wilson et al., 2005; Reanmongkol et al., 2006; Lin et al., 2010; Angel et al., 2014). About 31 *Curcuma* species have been studied, at which the most studied and relevant are turmeric (*C. longa*) and zedoary (*Curcuma zedoaria* (Christm.) Roscoe) (Dosoky and Setzer, 2018).

### Curcumin: A Historical Perspective

**Figure 1:**
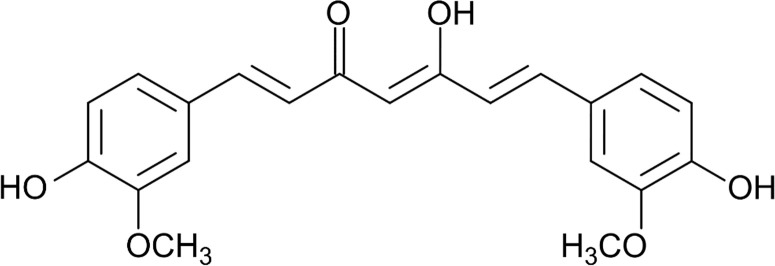
Chemical structure of curcumin.

The historic background of the *Curcuma* species begins in Far Eastern medicine and dates back 5,000 (Ayurveda) and 2,000 (Atharveda) years ago, respectively. *C. longa* contains different curcuminoids, although curcumin was found to be the most active one, first isolated in 1815 (Vogel and Pelletier, 1815), and the purified crystalline compound described in 1870 (Daube, 1870). The curcumin structure was first proposed by Polish scientists in 1910 (**Figure 1**) (Miłobędzka et al., 1910).

Although curcumin generally refers to 1,7-bis(4-hydroxy-3-methoxyphenyl)-1,6-heptadiene-3,5-dione, the compound is also known as “curcumin I”. In brief, curcumin is a diferuloylmethane with a crystalline yellow-orange colour, molecular weight of 368.39 g/mol, melting temperature of 183°C, and with the chemical formula C_21_H_20_O_6_. Chemically, it exhibits keto-enol tautomerism, i.e., it has a predominant keto form in neutral and acidic solutions, whereas the predominant form in the solid state and in an alkaline solution is its more stable enol form (Anand et al., 2007). There are two additional compounds known as curcumin, which are curcumin II [demethoxycurcumin, 1-(4-hydroxy-3-methoxyphenyl)-7-(4-hydroxyphenyl)-1,6-heptadiene-3,5-dione] and curcumin III [bisdemethoxycurcumin, 1,7-bis(4-hydroxyphenyl)-1,6-heptadiene-3,5-dione] (Buckingham, 2018).

Interestingly, this natural polyphenol is universally known as the “wonder drug of life” (Gera et al., 2017). In ancient times in the Far East, turmeric was used to treat inflammatory conditions of various organs, for liver and digestive tract problems, and on wound healing. In the 1970s, the first research on curcumin’s health benefits was carried out. In these and in later studies it was shown that curcumin has multiple therapeutic potentialities (Di Mario et al., 2007; Adhvaryu et al., 2008; Chandran and Goel, 2012; Yanpanitch et al., 2015; Gera et al., 2017; Salehi et al., 2019a). Nonetheless, turmeric was still not commercially considered as a therapeutic agent (Gera et al., 2017), and its use in medical clinics is rare because of its low bioavailability. The hydrophobic nature of curcumin after oral administration triggers a poor absorption rate by the gastrointestinal (GI) tract. On the other hand, curcumin seems to offer a promising potential for the therapeutic development from turmeric, categorized as a Generally Recognized As Safe (GRAS) material, with a stable metabolism and low toxicity (Nelson et al., 2017). Also worthy of note is the coloring attributes of curcumin for industrial applications (Joshi et al., 2009; Buckingham, 2018).

In this sense, this review focus on curcumin for food and biotechnological applications, health promotion, and disease prevention. Aspects related to curcumin’s bioavailability, bioefficacy, safety, side effects, and quality parameters are also addressed. A special emphasis is also given to curcumin’s multidimensional applications, food attractiveness optimization, agro-industrial procedures to offset its instability and low bioavailability, health concerns, and upcoming strategies for clinical application.

## Curcumin for Food and Biotechnological Applications

### Drifting From Colorant to Organoleptic Purposes (Organic Curcumin)

Turmeric has a long history of use as spice and food additive, widely used to ameliorate foodstuffs’ palatability and storage stability through its specific yellow color, taste, and antioxidant potential (Surojanametakul et al., 2010). The evaluation of the turmeric rhizomes’ organoleptic features revealed that they are yellowish in color, have an aromatic odor, and a slightly bitter taste (Duraisankar and Ravindran, 2015).

Curcumin is an orange–yellow dye practically insoluble in water and authorized by the European Union (EU) as a food additive. Other names, such as CI 75300, Natural Yellow 3 or diferuloylmethane, and the E code E100 are also used. Curcumin stability in aqueous solution is pH-dependent, with an optimum cut-off point ranging from pH 1–6. Its color turns to red in the charged state (pH<1 or pH>7) (Goel et al., 2008) and sunlight exposure accelerates curcumin degradation (Priyadarsini, 2009).

For nutritional purposes, curcumin is normally applied at a dose of 5-500 mg/kg, depending on the food category. It is mainly used in dairy products, beverages, cereals, mustard, food concentrates, pickles, sausages, confectionery, ice cream, and meat, fish, eggs, and bakery products (Lakshmi, 2014; Solymosi et al., 2015). Mixed with annatto, it is also added to seasonal sauces, mayonnaise sauces, and butter (Satyanarayana et al., 2010). Curcumin is a good and cheap alternative to saffron, although it cannot substitute the saffron taste, despite being named “Indian saffron” in Europe (Scartezzini and Speroni, 2000). As an additive, curcumin is stable during thermal treatment and in dry foods. It is relatively inert to reactions with other ingredients, although may form salts with phthalates and citrates, and it is inert in reactions with phosphates, chlorides, and bicarbonates (Stankovic, 2004).

An important issue regarding storage is the likelihood of microbial contamination that provokes foodstuffs’ deterioration and poisoning by food-borne pathogens (Ebrahimabadi et al., 2010), but many researchers have proven that curcumin exhibits some antimicrobial effects (Gupta and Ravishankar, 2005; Naz et al., 2010). For example, Liang et al. (2007) found that curcumin has good preservative effects on bread, bean curd, and cooked mutton. Gul and Bakht (2015) proved that chicken meal treated with curcumin-rich turmeric extract oil (1% or 2%) were safe and free from microbiological contamination over 90-day storage. Abdeldaiem (2014) showed that curcumin led to an increased oxidative stability of soy bean oil, and reduced total bacterial molds and yeast count in chicken breast fillet samples. Thus, curcumin suppressed lipid peroxidation and seemed to be useful as a natural preservative (Abdeldaiem, 2014). Jayaprakasha et al. (2006) also stated that linoleic acid oxidation was much lower in the presence of curcumin, and the antioxidant effect was about 80% when it was used as a dietary supplement. At the same dose, curcumin was able to double the resveratrol antioxidant activity, due to the double carboxyl and hydroxyl groups (Aftab and Vieira, 2010). To other foods, further studies are required to identify the best conditions of curcumin without interfering on food organoleptic properties.

### Looking at Food Industry Goals and Consumers’ Demands

Consumers’ concerns on the use of artificial additives in food products have markedly increased. Indeed, various surveys have indicated that people are requiring more data on the health effects of food additives (Tarnavölgyi, 2003).

Food coloring agents are used at both commercial and domestic levels, with an increasing amount of natural food coloring agents being commercially produced as synthetic dye alternatives in foods. This occurs partly due to consumers’ concerns about synthetic dyes, and on many regulatory bodies that have banned the use of some synthetic coloring agents (Jovičić et al., 2017). In addition, an increasing awareness among consumers is further fueling demand for curcumin over the forecast period, although it is also used in cosmetics and the pharmaceutical industry, where curcumin can be found in several forms, such as capsules, tablets, ointments, energy drinks, soaps, and in cosmetic products (Gupta et al., 2013).

**Figure 2:**
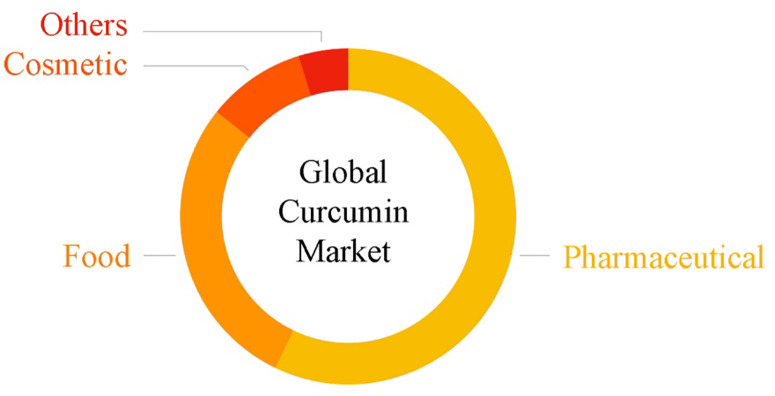
Global curcumin market by application.

The pharmaceutical industry, specially areas focused on anticancer drugs formulations, comprises the largest application segment, accounting for over 50% of the global market, followed by food and cosmetic industries (**Figure 2**). Modern cosmetology tries to apply valuable raw plant materials in cosmetics products’ manufacturing, and ayurvedic skin care products are projected to be a significant financial boost to the cosmetic market over the coming years. Curcumin is, nowadays, used as an active compound in skin care preparations due its remarkable antioxidant, anti-inflammatory, and anti-aging effects (Plianbangchang et al., 2007). Some of the significant cosmetic products include shampoos, oil serums, foundations, masques, conditioning lip balms, elixirs, and muscle gels. In addition, ultraviolet radiation exposure and rising environmental pollution is expected to boost the demand for skin care products containing curcumin.

According to a new study by Grand View Research, Inc., the global curcumin market size was worth over USD 46.6 million in 2016, with North America being the largest regional market in 2016, while India was one of the largest manufacturers of curcumin. Europe is expected to be the fastest growing region, with the market estimated to rise at a revenue-based compound annual growth rate (CAGR) of 14.8% over the forecast period, and the global market is expected to surpass USD 130 million by 2024. Rising scientific proficiency coupled with a large network of biotechnology and food chemistry applications is anticipated to increase the quality and quantity of curcumin, improving the future sales margins. Food applications will witness gains of 12.2% up to 2024, however, the presence of cheaper synthetic food products and substitutes may diminish the growth of the curcumin market.

### Current Legislation Practices

In 1975, curcumin was assessed by the Scientific Committee for Food (SCF) and its use as a food coloring was accepted without the need for further investigations, departing in this respect from the JECFA decision of a temporary acceptable daily intake (ADI) of 0-0.1 mg/kg B.W. (Commission of the European Communities, 1975). This temporary ADI was established based on existing ADI for turmeric oleoresin (0–2.5 mg/kg B.W.) and an average dose of 3% curcuminoids was assumed in turmeric. JECFA has also repeatedly evaluated the use of curcumin in 1974, 1978, 1980, 1982, 1987, 1990, 1992, 1995, 2000, 2002, and 2004. At its 44^th^ meeting in 1995, JECFA increased the temporary ADI to 0–1 mg/kg b.w. based on the no-observed-effect levels (NOEL) of 220 mg/kg b.w/day for hepatomegaly in the carcinogenicity study in mice, and a safety factor of 200 (Forty-fourth report of the joint FAO/WHO Expert Committee on Food Additives, 1995). The new ADI was extended, pending submission of reproductive toxicity results with curcumin. The results of the multigeneration study in rats fed with curcumin for 24 weeks were available to the Committee for evaluation at its 61^st^ meeting in 2004 (Sixty-first report of the joint FAO/WHO Expert Committee on Food Additives, 1995). The Committee highlighted that the previous temporary ADI came from a study on turmeric oleoresin (79-85% curcuminoids) and allocated a new ADI of 0-3 mg/kg b.w. for curcumin based on the NOEL of 250-320 mg/kg b.w/day in the multigenerational study in rats, and application of a safety factor of 100 (Sixty-first report of the joint FAO/WHO Expert Committee on Food Additives, 1995). Ethyl acetate with a residual limit of 50 mg/kg and carbon dioxide as a supercritical fluid were added as alternative solvents (Sixty-first report of the joint FAO/WHO Expert Committee on Food Additives, 1995). At last, the Committee established that there was no adequate data available to assess exposure, and an addendum with a toxicological monograph and a chemical and technical assessment (CTA) were thus prepared (Sixty-first report of the joint FAO/WHO Expert Committee on Food Additives, 1995).

In 2010, the Panel on Food Additives and Nutrient Sources agreed with JECFA that curcumin is neither carcinogenic nor genotoxic (EFSA Panel on Food Additives and Nutrient Sources added to Food (ANS), 2010). The Panel concluded that, in eleven European countries, the intake estimates for children (1-10-year old) are above the ADI from both naturally-occurring curcumin in foods and through the form of dye (EFSA Panel on Food Additives and Nutrient Sources added to Food (ANS), 2010). The Panel also noted that curcumin intake in foods (as spice and in curry powder) amounts to <7% of the ADI of 3 mg/kg b.w./day for adults and older individuals (EFSA Panel on Food Additives and Nutrient Sources added to Food (ANS), 2010). A refined exposure assessment was then performed by the European Food Safety Authority (EFSA) using new usage data from the industry, and concluded that for adolescents, adults, and older individuals, the exposure estimates were lower than those reported by the ANS Panel in 2010 at both mean and high levels (95^th^ percentile) of exposure (European Food Safety Authority, 2014). Using the refined estimated exposure scenarios, low exposure estimates at mean levels of exposure were found in toddlers and children, while these levels exceeded the ADI at the high level (95^th^ percentile) of exposure, with the main contributing food categories for all scenarios being flavored drinks and fine bakery wares (European Food Safety Authority, 2014).

Regarding curcumin purity, it was established as not less than 90% total coloring matters. The residual 10% was specified according to the Commission Directive 2008/128/EC and JECFA. The maximum allowed lead concentration is given as ≤2 mg/kg and, with regard to other metals, in 2008, EFSA established a Tolerable Weekly Intake (TWI) of 1 mg aluminum/kg b.w/week (European Food Safety Authority, 2008).

Six turmeric-related monographs appeared in USP35–NF30 Pharmacopeial Forum (PF) 33(6), Nov-Dec, 2007. These monographs contain validated and specific analytical methods to ensure article identity, and to protect consumers and industries from low-quality and adulterated products. Rhizoma *Curcumae Longae* appeared in the WHO Monographs on Selected Medicinal Plants, Volume 1 (WHO, 1999); it is used in the European market as listed in the European Pharmacopoeia monograph #2543, and the European Scientific Cooperative on Phytotherapy (ESCOP) included it in its 2^nd^ Edition Supplement monographs, 2009 (ESCOP, 2009).

Finally, it should be noted that while turmeric essential oils and oleoresins have a GRAS status, curcumin is not on any readily accessible U.S. Food and Drug Administration (FDA) GRAS list (2016). The FDA concluded that curcuminoids used as antioxidant and flavoring agents at maximum levels of >20 mg/serving in specific foods are safe. However, the agency *“has not, however, made its own determination regarding the GRAS status of the subject use of curcuminoids”* (2013).

## Curcumin on Health Promotion and Disease Prevention

**Figure 3:**
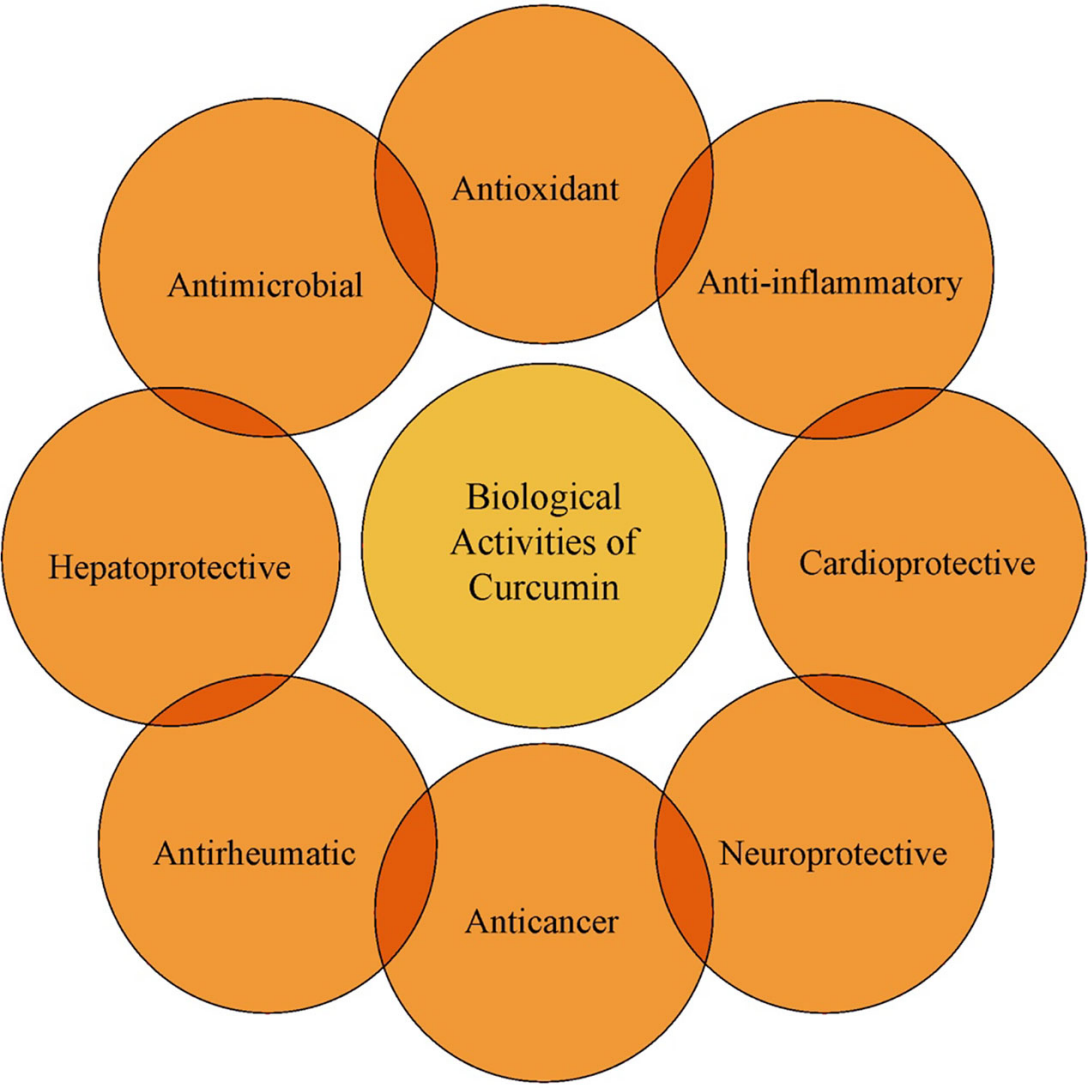
Schematic illustration of curcumin biological activities.

Multiple biological effects on both health promotion and disease prevention have been recognized in curcumin and its derivatives (**Figure 3** **)**. Indeed, a bibliometric analysis performed by Yeung et al. (2019) revealed that the United States, China, India, Japan, and South Korea are the main contributors to the scientific advances found on curcumin bioactive effects, with the most focused being their anticancer, inflammatory, and antioxidant potential, as already stated by Xu et al. (2018). In the following sections, the pre-clinical and clinical data related to curcumin bioactive effects is briefly discussed and the respective mode of action cleared.

### Antioxidant Activity

Curcumin antioxidant effects have been the most widely explored in the literature. Various *in vitro* and *in vivo* studies have been conducted, and the antioxidant potential of curcumin has been attributed to its chemical structure, including carbon-carbon double bonds, β-diketo group and phenyl rings with hydroxyl, and *o*-methoxy groups (Wright, 2002; Priyadarsini et al., 2003; Menon and Sudheer, 2007). Many mechanisms can explain the antioxidant activity as binding free radicals, hydrogen atom donors, and electron donors to neutralize free radicals. For that, laser flash photolysis and pulse radiolysis have been used to elucidate the mechanism of action of curcumin’s antioxidant activity (Jovanovic et al., 1999; Nardo et al., 2008).

Curcumin is able to promote its antioxidant activity by scavenging a variety of reactive oxygen species (ROS) as superoxide radicals, hydrogen peroxide, and nitric oxide (NO) radicals and by inhibiting lipid peroxidation (Ak and Gulcin, 2008). This latter activity is due to the enhancement of many antioxidant enzymes activity, such as SOD, CAT, GPx, and OH-1. Curcumin can also increase the GSH levels by upregulating glutathione transferase and their mRNAs. Curcumin can also inhibit ROS-generating enzymes, such as LOX, COX, and xanthine oxidase. Curcumin is also considered a chain-breaking antioxidant because of its lipophilic nature, potentially acting as a peroxyl radicals scavenger (Priyadarsini et al., 2003).

### Anti-Inflammatory Activity

**Figure 4:**
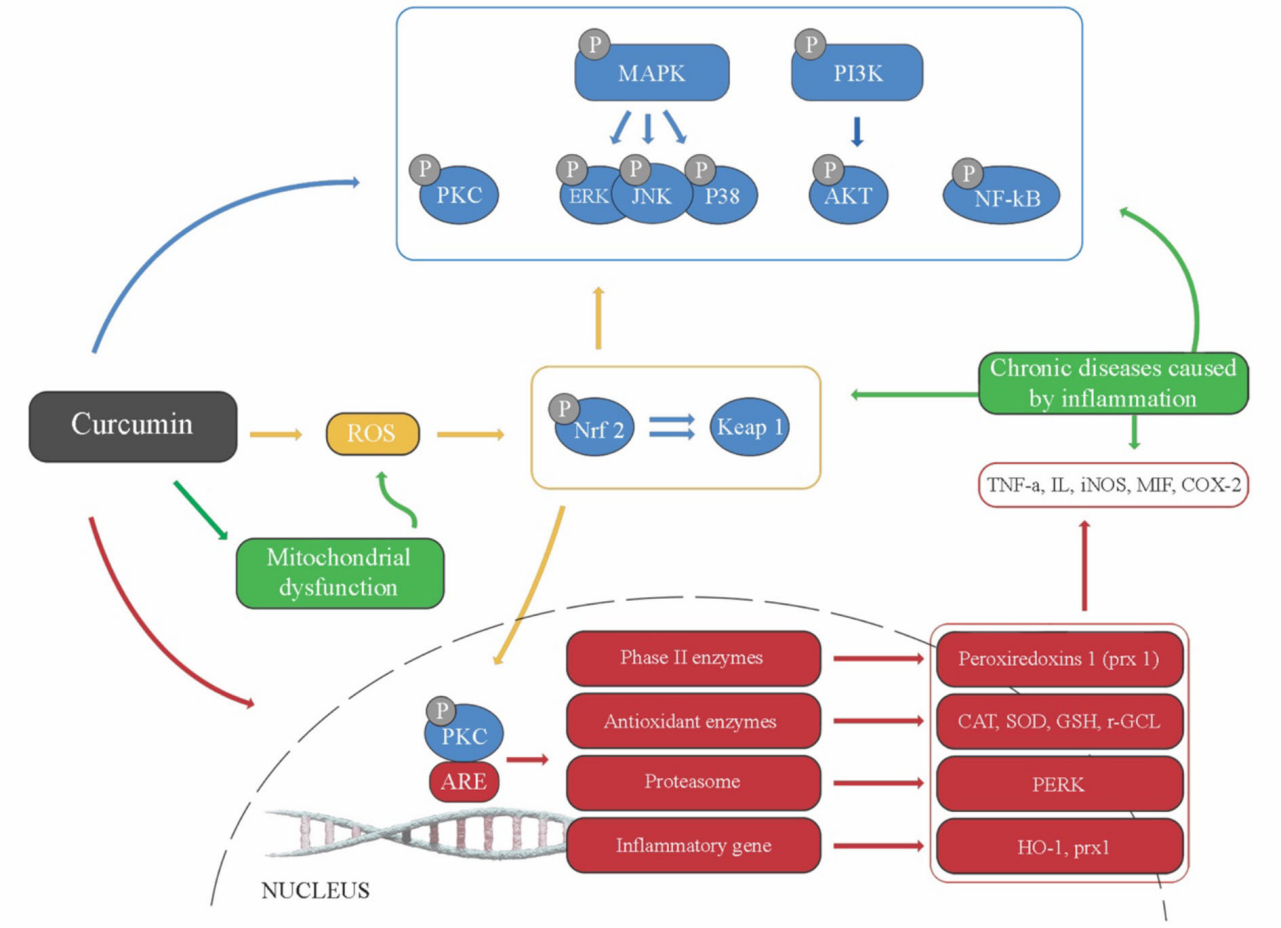
Curcumin anti-inflammatory mechanisms (adapted from He et al., 2015).

In the literature, numerous *in vitro* and *in vivo* studies have shown that curcumin has a great potential for treating numerous inflammatory diseases (Aggarwal and Sung, 2009; Cianciulli et al., 2016; Edwards et al., 2017; Dai et al., 2018). It was shown that curcumin can: i) Inhibit pro-inflammatory transcription factors (NF-κB and AP-1); ii) Reduce the pro-inflammatory cytokines TNFα, IL-1b, IL-2, IL-6, IL-8, MIP-1a, MCP-1, CRP, and PGE2; iii) Down-regulate enzymes such as 5-lipoxygenase and COX-2 and -5; and iv) Inhibit the mitogen-activated protein kinases (MAPK) and pathways involved in nitric oxide synthase (NOS) enzymes synthesis (Aggarwal and Sung, 2009; Panahi et al., 2014a; Panahi et al., 2014b; He et al., 2015; Machova Urdzikova et al., 2015). The anti-inflammatory mechanisms are shown in **Figure 4**.

On the other side, and given that oxidative stress triggers chronic inflammation, a close relationship between antioxidant molecules and its anti-inflammatory potential is becoming increasingly clear. In this way, curcumin is also able to modulate the NF-κB expression. In fact, the NF-κB pathway activation leads to proinflammatory cytokine production, such as interleukin (IL-1, IL-2, IL-6, IL-8) and TNFα, known to be responsible for the activation of pro-inflammatory signaling pathways. In addition, curcumin could decrease the oxidative stress and inflammation through the Nrf2 pathway. The COX pathway leads to the conversion of arachidonic acid into prostaglandins and thromboxanes, with two COX isoenzymes (COX-1 and COX-2) being involved. Particularly, COX-2 is induced by various cytokines and tumor promoters, thus closely linked to inflammation and carcinogenesis, with many studies demonstrating that curcumin can inhibit the induction of COX-2 gene expression (Yang et al., 2017).

### Neuroprotective Effect

Neurodegenerative disorders - such as Parkinson disease (PD) and Alzheimer’s disease (AD) - major depression, and epilepsy affect millions of people worldwide, with an increasing incidence rate.

**Figure 5:**
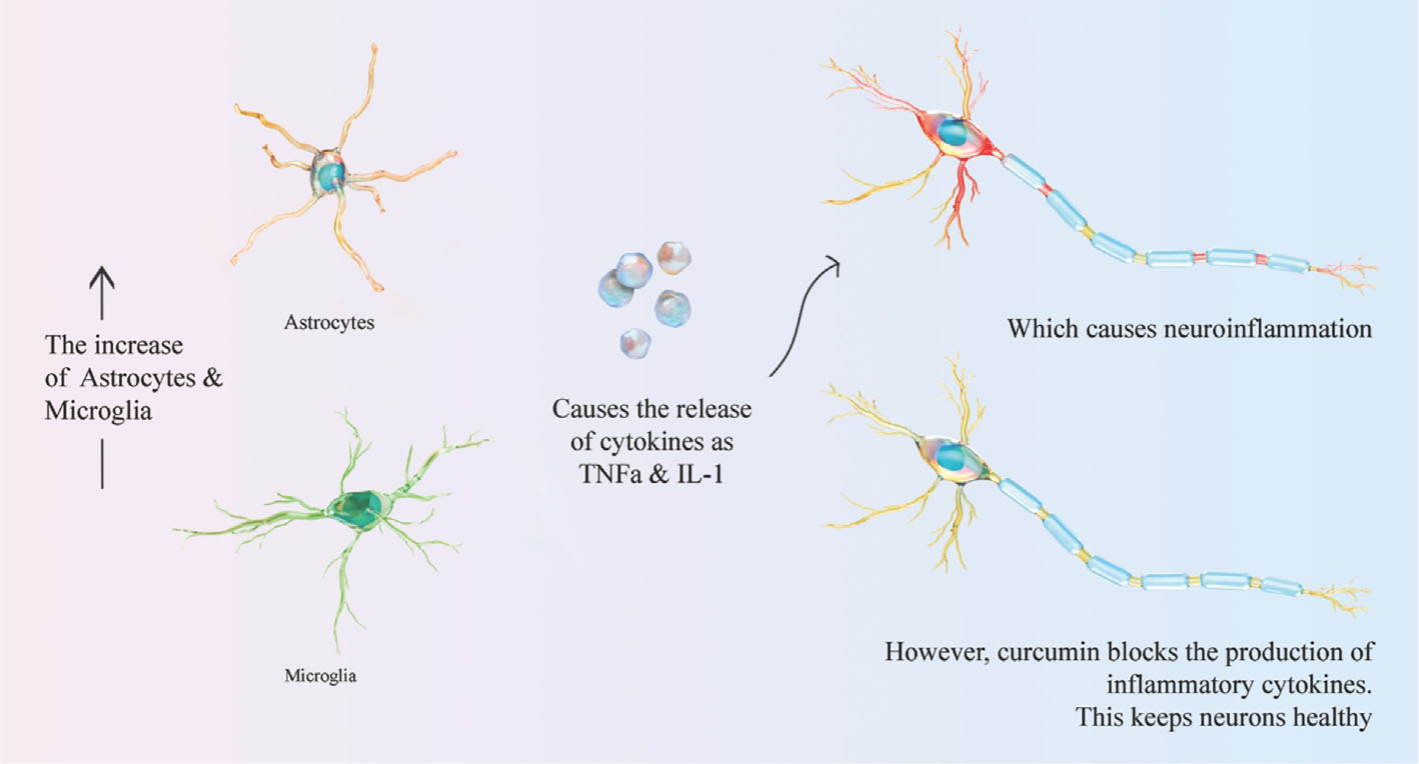
Curcumin mechanism of action in neuroinflammation.

Neuroinflammation is a chronic inflammation that leads to neuronal metabolism changes that result in neuronal degradation. In neuroinflammatory states, the neuronal death is increased by microglia and astrocytes activation. The latter are responsible for proinflammatory cytokines’ release, such as TNFα and IL-1. Based on existing studies, curcumin has been used as a potential therapeutic agent for various neurological disorders, such as dementia, AD, PD, multiple sclerosis, and Huntington’s disease (HD), due its antioxidant, anti-inflammatory, and anti-protein aggregating abilities (Ye and Zhang, 2012; Wu et al., 2013; Song et al., 2016; Teter et al., 2019; Salehi et al., 2020a). **Table 1** shows the neuroprotective effect of curcumin and its related mechanism. For example, it has been shown that curcumin blocks the inflammatory cytokines and prostaglandins production in activated microglia and astrocytes (Yang et al., 2014; Cianciulli et al., 2016). It also decreases the production of TNFα, IL-1β, macrophage inflammatory protein (MIP-1β), monocyte chemoattractant protein (MCP-1), and IL-8 in microglial and astrocytes cells (Chen et al., 2014). These aspects are briefly shown in **Figure 5**.

**Table 1:** Curcumin and neuroprotective mechanisms.

Study type	Subjects	Dose/frequency	Outcome & Mechanisms	Reference
*In vivo*	Male Sprague–Dawley rats	300 mg/kg b.w.	Reducing oxidative stress levels in middle cerebral artery occlusion	(Wu et al., 2013)
			Increasing phospho-Akt, Nrf2, and NQO1 expression levels	
			Upregulating Nrf2 activity	
*In vivo*	Male Sprague–Dawley rats	5, 10 and 20 mg/kg /day for 30 days	Upregulating PI3K expression	(Yang et al., 2014)
			Activating the BDNF/TrkB-dependent pathway	
			Increasing the contents of monoaminergic neurotransmitters	
*In vitro / In vivo*	Primary hippocampus neurons/APP/PS1 transgenic mice	150 mg/kg /day for 4 weeks	Reducing the activation of microglia and astrocytes	(Liu et al., 2016)
			Inhibiting the NF-κB signaling pathway	
			Increasing the transcriptional activity and protein levels of PPARγ	
*In vivo*	Male Sprague–Dawley rats	5, 10 and 20 mg/kg /day for 30 days	Increasing levels of SOD and GSH-Px	(Song et al., 2016)
			Increasing levels of Dopamine and acetylcholine	
			Upregulating of bFGF, NGF, and TrkA	
*In vivo*	Male Wistar rats	100 mg/kg b.w. for 28 days	Modulating the PI3K/Akt/GSK3β neuronal survival pathway	(Srivastava et al., 2018)
			Increasing levels of pCaMKIIα/CaMKIIα and PSD95	
			Increasing levels of pCREB/CREB	
*In vivo*	Tg2576 mice	160 and 5000 mg/mL for 6 months	Immunomodulator of the TREM2-CD33-TyroBP hub	(Teter et al., 2019)
			Stimulating phagocytosis and altering inflammatory cytokines expression	
			Reducing levels of miR-155	
*In vivo*	Male Sprague Dawley rats	200 mg/kg b.w.	Attenuating autophagic activities through mediating the PI3K/Akt/mTOR pathway	(Huang et al., 2018)
			Suppressing an inflammatory reaction by regulating the TLR4/p38/MAPK pathway	
*In vivo*	Male Sprague Dawley rats	100 mg/kg b.w.	Activating the Nrf2-ARE pathway	(Dai et al., 2018)

**Figure 6:**
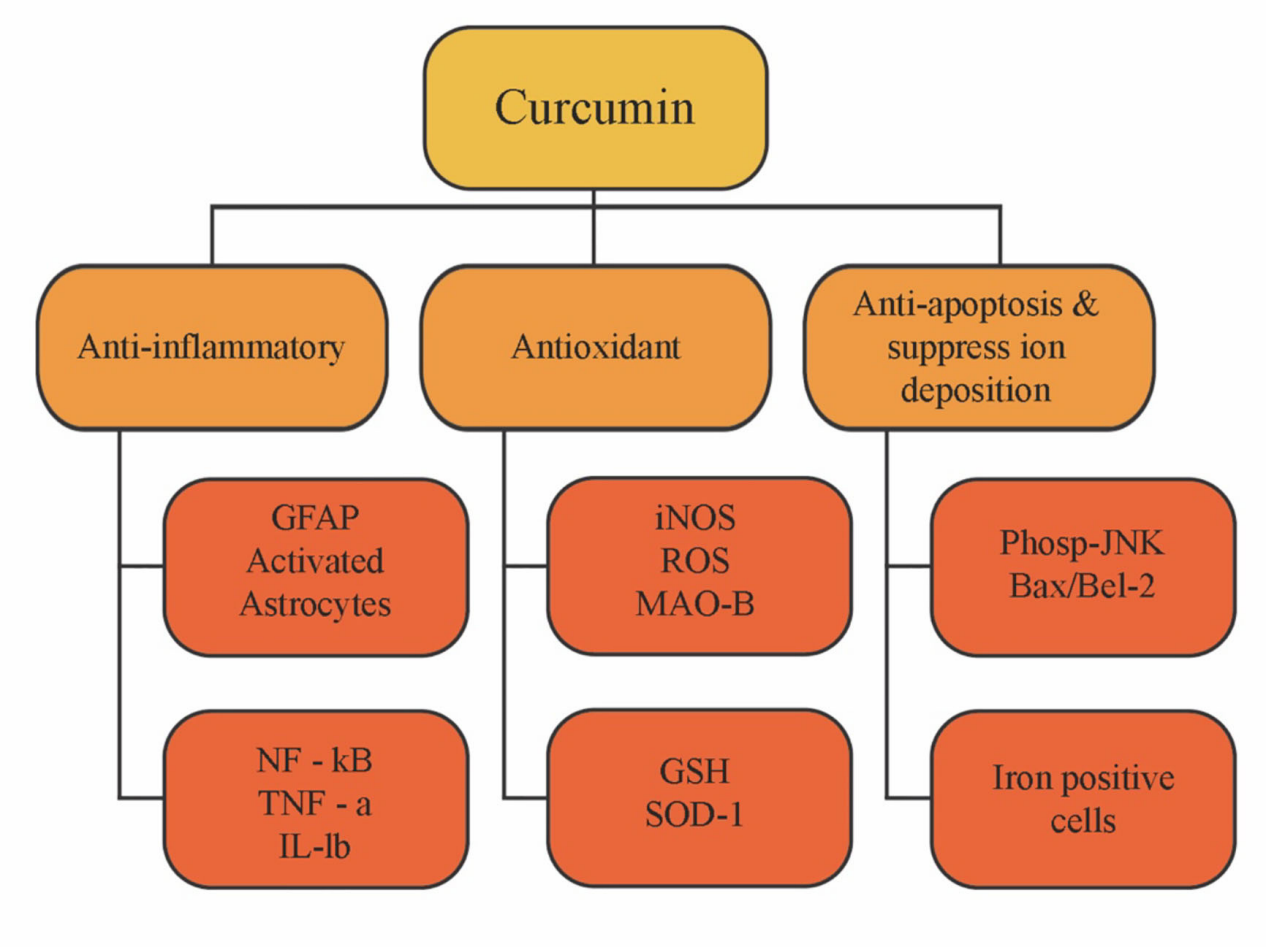
Neuroprotective mechanisms of curcumin in treating Parkinson’s disease (adapted from Wang et al., 2017).

In AD, the main cause is the deposit of Amyloid-ß (Aß) peptides plaques, which is the consequence of microgliosis, astrocytosis, and the existence of proinflammatory substances in the brain. Curcumin was found to reduce AD symptoms by (Goozee et al., 2016; Ganesh et al., 2017): 1) Inhibiting the Aß peptide production by altering the amyloid precursor protein trafficking; 2) Binding the Aß peptides and influencing their aggregation; 3) Attenuating the hyperphosphorylation of tau and enhancing its clearance; 4) Reducing Aß induced toxicity through the inhibition of JNK-3 phosphorylation; 5) Lowering cholesterol levels which reduces AD risk; 6) Protecting the blood-brain barrier by up-regulating OH-1 expression; 7) Inhibiting acetylcholinesterase; 8) Playing a role in cell signaling through activating Wnt pathways; and 9) Reducing inflammation and oxidative damage. In the same way, some studies have shown that PD can be treated with curcumin. **Figure 6** shows the neuroprotective mechanisms of curcumin in treating PD.

In multiple sclerosis, an autoimmune inflammatory disease that primarily affects young adults and women through demyelinating lesions, curcumin has also shown neuroprotective effects through different mechanisms, including antioxidant, anti-inflammatory, and anti-proliferative mechanisms. Curcumin was also able to modulate several molecular targets, such as transcriptional factors (NF-κB, Nrf2, AP-1, STAT-1,-3,-4), enzymes (COX-2, iNOS, OH-1, LOX, XO), inflammatory cytokines (chemokine ligand, interleukin, TNFα), proteins (caspase-3,-9, Bcl-2, Prostaglandin, CRP, myosin light chain), protein kinase (AK, JNK, JAK, MAPK) and growth factors and receptors (TLRs, chemokine receptor, TGF-α, TGF-ß) (Qureshi et al., 2018).

### Anticancer Effect

**Figure 7:**
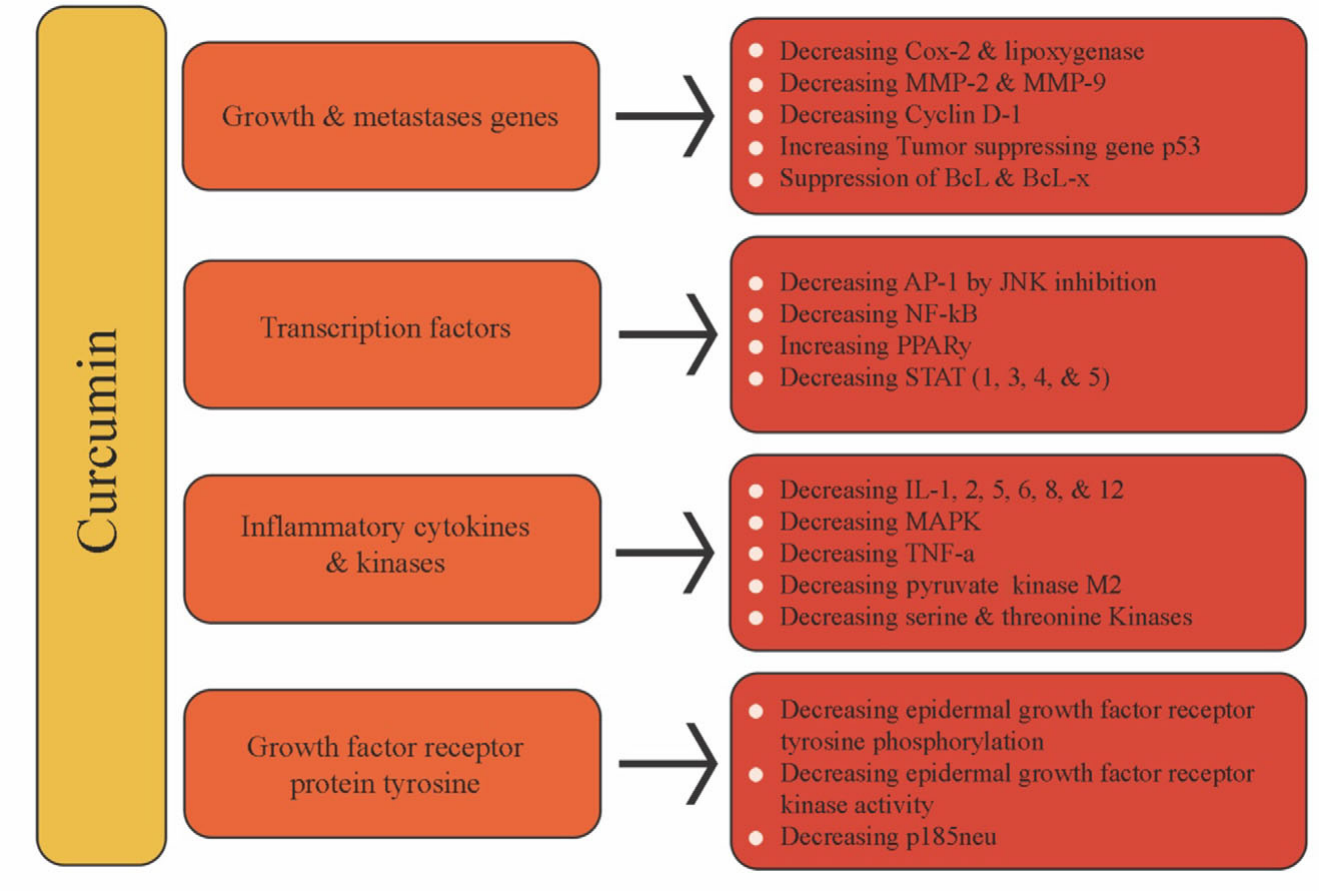
Curcumin molecular targets in cancer cells.

In the literature, extensive preclinical studies can be found assessing curcumin’s anticancer effect, with increasing attention being given to its related mechanism of action (**Table 2**). Curcumin has been shown to prevent carcinogenesis by affecting two processes: angiogenesis and cancer cell growth. It also suppresses cancer cell metastasis and induces cancer cell apoptosis. The different molecular targets through which curcumin acts, downregulating or upregulating, is shown in **Figure 7**.

**Table 2:** Curcumin anticancer effect and mechanisms.

Study type	Subjects	Dose / frequency	Outcome & Mechanisms	Reference
*In vitro*	MCF-7 breast cancer cell line	50 µg/mL	Decreasing *Mcl-1* gene expression	(Khazaei Koohpar et al., 2015)
			Inducing apoptosis	
*In vitro*	HNSCC cells (FaDu & Cal27)	12.5 µM	Increasing pro-apoptotic protein Bik and Bim	(Xi et al., 2015)
			Reducing phosphorylation of NF-κB and STAT-3	
			Suppressing cyclin D1 and D2 expression	
*In vitro*	MCF-7 breast cancer cell line	2.5 µM	Inducing Bcl-2 expression (apoptosis)	(Zhan et al., 2014)
			Suppression of the EGFR expression	
*In vitro*	MCF-7 and MDA-MB-231 breast cancer cells	2–10 μM	Activating the ERK signaling pathway	(Wang et al., 2016b)
			Autophagy induced by activation of JNK	
*In vitro*	MCF7, MDA-MB-231, and SKBR3 breast cancer cells		Increasing *Tusc7* and *GAS5* expression	(Esmatabadi et al., 2018)
*In vitro*	MDA-MB-231 breast cancer cell	40 µM	Activating p38-MAPK	(Meena et al., 2017)
			Decreasing CDK2, CDK4, cyclin D1, and cyclin E levels	
			Inducing cell cycle arrest at G1/ and G2/M phases	
*In vitro*	Patu8988 pancreatic cell line	10, 15 and 20 μM	Suppressing cell growth, inhibiting migration and invasion, and inducing apoptosis	(Zhou et al., 2016)
			Downregulating YAP and TAZ expression	
			Suppressing *Notch-1* expression.	
*In vitro*	PANC1 and BxPC3 cell lines	10 - 80 µg/mL	Inducing cell cycle arrest at the G2/M phase	(Zhu and Bu, 2017)
			Upregulating of Bax and LC3II expression	
			Downregulating Bcl2 expression	
*In vitro*	HCT116 colon cancer cell line	5, 10 and 20 μM	Inhibiting EIF2, eIF4/p70S6K, and mTOR signaling pathways	(Wang et al., 2016a)
			Inhibiting *de novo* protein synthesis	
			Increasing ROS levels due to mitochondrial dysfunction	
*In vitro / In vivo*	SW480 colon cancer cell line	200 mg/kg b.w. for 5 days	Decreasing β-catenin expression	(Dou et al., 2017)
			Upregulating of Nkd2	
			Suppressing the Wnt/β-Catenin Pathway via miR-130a	
*In vivo*	Male Sprague–Dawley rats	25, 50, and 75 mg/kg b.w.	Downregulating the PI3-K/Akt/PTEN pathway	(Rana et al., 2015)
			Increasing pro-apoptotic Bad and Bax expression	
			Inhibiting Bcl2 expression	
*In vivo*	Male nude BALB/c mice	100 mg/kg b.w. each 2 days	Downregulating Notch and HIF-1 mRNA expression	(Li et al., 2018b)
			Suppressing VEGF and NF-κB expression VEGF and NF-κB expression	
*In vitro*	Human lung cancer cells (NCI-H1299, NCI-H460, NCI-H520 and NCI-H446)	5-40 µM	Upregulating IGFBP-1	(Man et al., 2018)
			Suppressing the PCNA and NF-κB pathway	
			Activating JNK phosphorylation	
*In vitro / In vivo*	HCT11 and HT29 colon cancer cells Male nude BALB/c mice	10, 20, 30 and 40 µM, 40 mg/kg b.w.	Downregulating NF-κB activation	(Zhang et al., 2017)
			Inhibiting AMPK/ULK1-dependent autophagy	
*In vitro*	Mouse prostate cancer cells TRAMP-C1	50 and 100 nM	Activating Nrf2 expression	(Li et al., 2018a)
			Reducing the methylation rate of the Nrf2 promoter	
			Reducing H3k27me3 enrichment on the Nrf2 promoter region	

The role of angiogenesis in cancer is well-known. In fact, cancer cells can produce new blood vessels by proangiogenic factors stimulation. Curcumin has been shown to have anti-angiogenic activity by inhibiting angiogenic factors stimulators, as VEGF and basic fibroblast growth factor. In fact, curcumin has revealed to be able to downregulate the VEGF expression through NF-κB and AP-1 regulation, attenuating IL-8 expression (Yance and Sagar, 2006). Astinfeshan et al. (2019) showed that curcumin can inhibit angiogenesis through VEFGR and the PI3K/Akt signaling pathway modulation. Moreover, it was shown that curcumin can downregulate MMP-2 and MMP-9 and upregulate the tissue inhibitor metalloproteinase-1, which insures extra cellular matrix stability and coherence (Yance and Sagar, 2006).

Curcumin can also induce apoptosis in cancer cells through a p53-dependent pathway. p53 is known to be one of the most important tumor suppressor proteins, affecting cell proliferation apoptosis and DNA damage (Kandoth et al., 2013). Several studies have revealed an interplay between p53 and cancer-related miRNAs (Hermeking, 2007; Ye et al., 2015). Ye et al. (2015) also showed that the curcumin proapoptotic effect depends on miR-192-5p and miR-215, which activates the p53 in non-small cell lung cancer. Other studies have shown that curcumin-triggered apoptosis is p53-independent in HT-29 colon cancer cells (Watson et al., 2010).

Cyclin dependent kinase (CDKs) are serine/threonine kinases that form a complex with their respective cyclin partner, thus controlling cell cycle progression. Altered CDKs’ expressions are always observed in cancer cells. Chiu and Su (2009) showed that treating triple negative breast cancer cell line MDA-MB-231 with curcumin led to a disruption in CDKs/cyclin complexes, necessary for cell cycle progression and down-regulation of cyclin D1, required for progression through the G1/S phase, and whose overexpression is associated with most breast cancers, thus leading to cell cycle arrest at G1.

Curcumin has been proposed as highly effective against Ras-overexpressed cancer conditions. In fact, Cao et al. (2015) showed that curcumin inhibits AGS gastric cancer cells proliferation by downregulating the Ras proteins and upregulating ERK. Banerjee et al. (2017) demonstrated that curcumin-based intervention shifts the oncogenic RAS-induced MEK/ERK pro-proliferative pathway toward p38MAPK/JNK1 pro-death signaling. Curcumin also revealed to be able to inhibit and downregulate the PI3K/Akt signal pathway in cancer models (Rana et al., 2015; Kasi et al., 2016). In the same line, targeting the Wnt/ß-catenin signaling pathway is a promising approach in cancer therapy. In fact, Wnt/ß-catenin overexpression is implicated in human cancers, with curcumin being able to trigger cell cycle arrest at G_2_/M phase through modulation of Wnt/ß-catenin signaling pathway. Dou et al. (2017) also showed that curcumin is able to inhibit colon cancer by Wnt/β-catenin pathways suppression *via* miR-130a.

With its anticancer effect, curcumin can target cancer transcription factors. Many studies have shown that it can block the NF-κB and AP-1 families of transcriptional factors (Mishra et al., 2015; Man et al., 2018). Marquardt et al. (2015) have tested the effect of curcumin in curcumin-sensitive and curcumin-resistant liver cancer cell lines. The authors found that in curcumin-sensitive cells, NF-κB was inhibited, while curcumin-resistant cells retained the NF-κB function. In lung cancer cells, curcumin decreased the expression of proliferating cell nuclear antigen (PCNA), p‐PI3K, and NF‐κB (Man et al., 2018). The sensitivity of human and rat glioma cells to radiation increased following curcumin treatment, and both AP-1 and NF-κB expression were inhibited (Shanmugam et al., 2015). Also, curcumin has been shown to suppress STAT expression. Curcumin was able to decrease the expression levels of STAT-3-regulated cyclin D1, BCL-2, and Bcl-xL in pancreatic cancer cells (Rajitha and Nagaraju, 2017). Shanmugam et al. (2015) showed that curcumin could inhibit IL-6 induced STAT-3 phosphorylation and STAT-3 nuclear translocation in multiple myeloma. Moreover, curcumin was also able to inhibit cell proliferation, migration, and invasion, but promoted apoptosis in retinoblastoma cells, with their antitumor activities appearing to be *via* the up-regulation of miR-99a, and thereby the inhibition of the JAK/STAT pathway.

### Hepatoprotective Effect

Several agents, such as alcohol, drugs, pollutants, parasites, and dietary components, among others, can trigger acute and chronic liver injuries, including liver fibrosis, non-alcoholic steatohepatitis, non-alcoholic liver disease, and even cirrhosis. Curcumin has been extensively studied for its hepatoprotective effects (Rahmani et al., 2016; Tung et al., 2017; Peng et al., 2018; Macías-Pérez et al., 2019).

Choudhury et al. (2016) demonstrated that a curcumin injection (8.98 µM) in Swiss albino rats with CCl_4_-induced hepatotoxicity decreased the NADH oxidase level and increased the GR and GST levels and succinate dehydrogenase activity. For the same type of hepatotoxicity, curcumin administration (200 mg/kg) in Sprague-Dawley rats increased the hepatic glutathione level and decreased the lipid peroxidase level and the activities of both alanine transaminase (ALT) and aspartate aminotransferase (AST) (Lee et al., 2016). So, curcumin may be a promising agent to prevent oxidative stress-related liver disorder, by decreasing ALT, AST, and alkaline phosphatase levels, increasing GST, GR, GPx, SOD and CAT, and reducing NO as well as inhibiting ROS production (Farzaei et al., 2018). Furthermore, Badria et al. (2015) showed that curcumin treatment increased the endogenous antioxidant levels (ascorbic acid, GSH, SOD, and CAT) in the liver in chronic iron overloaded male rats.

In case of drug-induced hepatoxicity, such as that triggered by streptozotocin and paracetamol abuse, curcumin was able to attenuate such effects in mice. Afrin et al. (2015) found that curcumin administration in Sprague Dawley rats with streptozotocin-induced diabetes inhibited TNFα, IL-1ß, MAPK, and apoptosis signal-regulating kinase 1 (ASK1) in liver tissues. In non-alcoholic steatohepatitis induced by low-dose streptozotocin and a high-fat diet, Afrin et al. (2017) found that curcumin treatment was able to decrease oxidative stress, inflammation, and lipogenesis, and attenuated fibrosis and HMGB_1_-NF-κB translocation and signaling. Concerning paracetamol-induced hepatotoxicity, curcumin administration attenuated mitochondrial dysfunction by scavenging free radicals, induced antioxidant enzymes expression, and inhibited NF-kB and transient receptor potential melastatin 2 (TRPM2) channels (Granados-Castro et al., 2016; Kheradpezhouh et al., 2016).

In mice with alcoholic fatty liver, curcumin administration attenuated hepatocyte necroptosis, suppressed the ethanol-induced pathway, inhibited glyoxylate, dicarboxylate, and pyruvate metabolisms, modulated antioxidant signaling pathways and upregulated detoxifying genes expression *via* the ERK/p38-MAPK pathway (Xiong et al., 2015; Lu et al., 2016; Guo et al., 2017).

It has also been demonstrated that curcumin can attenuate liver fibrosis and cirrhosis (Chen et al., 2014; Zhong et al., 2016). Curcumin administration in Sprague-Dawley rats with CCl_4_-induced hepatic fibrosis led to a decrease in liver fibrosis through: i) reducing extracellular matrix overproduction in HSCs; ii) disrupting PDGF-R/ERK and mTOR pathways; iii) activating PPAR-γ; iv) upregulating PTEN and miR-29b expression; and v) downregulating cannabinoid receptors (CBR) type 1 and DNA methyltranferase 3b (Chen et al., 2014; Zhang et al., 2014).

### Cardioprotective Effect

**Figure 8:**
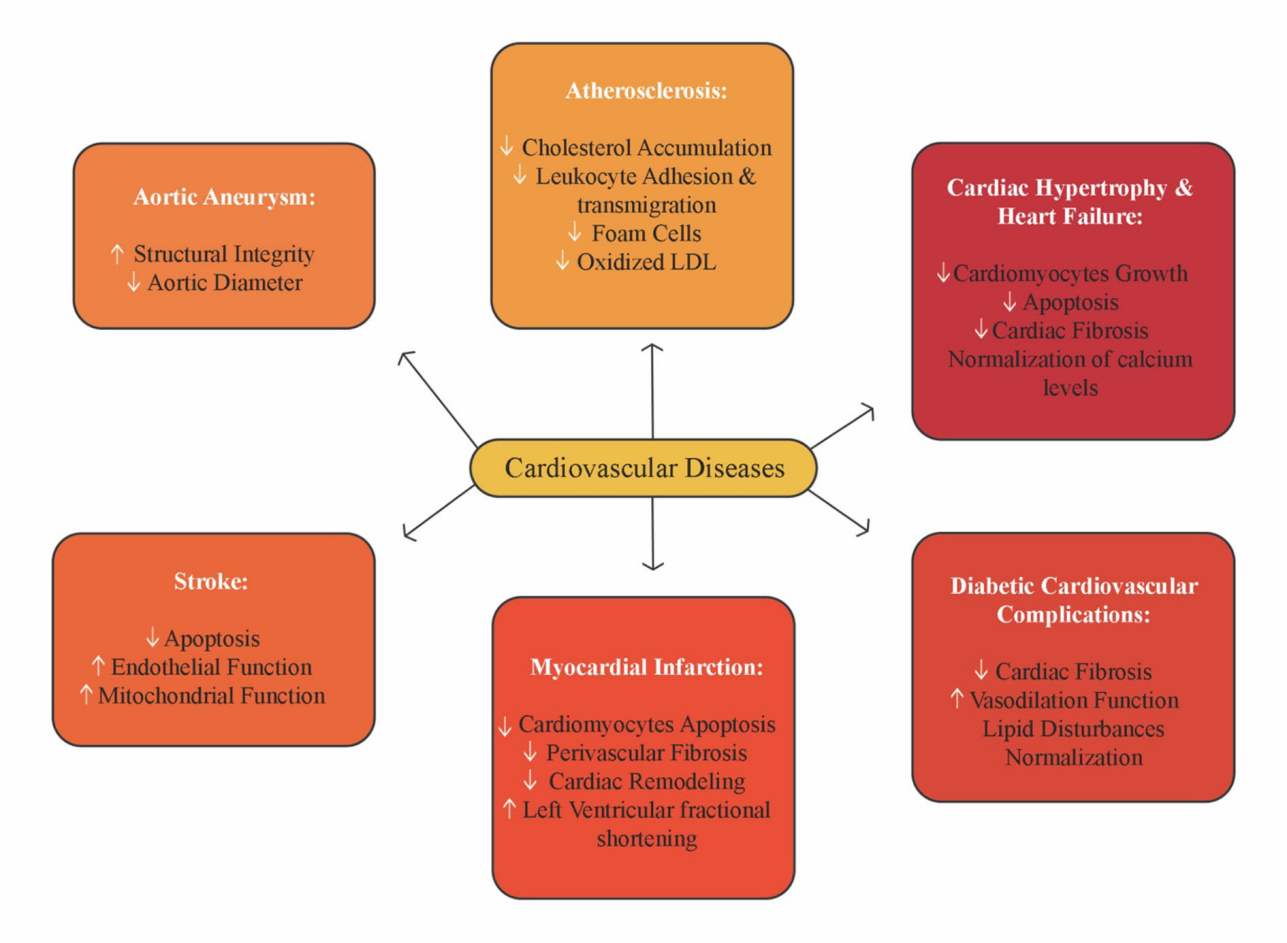
Curcumin action on cardiovascular diseases (adapted from Li et al., 2019).

Cardiovascular (CV) diseases are considered a worldwide human health threat and are associated with high morbimortality rates. Studies have shown that curcumin is effective in protecting from CV diseases (Jiang et al., 2017; Li et al., 2019). **Figure 8** illustrates the mechanism of action of curcumin in CV diseases. As seen, curcumin CV benefits are mostly related to their protective effects on atherosclerosis, cardiac hypertrophy, heart failure, aortic aneurysm, stroke, myocardial infarction, and diabetic CV complications (Salehi et al., 2020b). As main molecular targets, it is noteworthy that curcumin activates the Nrf2 which leads to HO-1 induction, responsible for cytoprotective and anti-inflammatory effects against oxidative stress (Pittala et al., 2018). For example, Sunagawa et al. (2018) showed that curcumin is a natural p300 histone acetyltransferase (HAT) inhibitor, while Monfoulet et al. (2017) reported that curcumin is able to enhance endothelial function and to decrease the TNFα-induced monocyte adhesion in endothelial cells through NF-κB inhibition. Furthermore, Yao et al. (2016) found that curcumin reduces angiotensin II type 1 receptor (AT1R) expression, thus preventing CV diseases. Effectively, curcumin decreases the binding potential of AT1R gene promoter with the specificity protein 1 (SP1). Cao et al. (2018) demonstrated that curcumin may attenuate chronic heart failure by increasing p38 MAPK, JNK, and ASK1.

## Curcumin Bioavailability and Bioefficacy: A Brushstroke

Scientific evidence has shown that curcumin exhibits antioxidant, hypoglycemic, wound healing, anti-inflammatory (including psoriasis-like inflammation), anti-asthmatic, antiviral, antimicrobial, antifungal, anticancer, chemo-sensitization, and radio-sensitization effects (Cheng et al., 2001; Garcea et al., 2005; Goel and Aggarwal, 2010; Shahani et al., 2010; Gou et al., 2011; Wang et al., 2012; Chang et al., 2013; Dovigo et al., 2013; Phillips et al., 2013; Subhashini et al., 2013; Sun et al., 2013a; Karlowicz-Bodalska et al., 2017; Salehi et al., 2019b), as well as being able to reduce the pro-fibrotic effects in idiopathic pulmonary fibrosis, to prevent intra-cerebral hemorrhage, and to ameliorate lipopolysaccharide induced cardiac hypertrophy (Smith et al., 2010b; Sun et al., 2011; Chowdhury et al., 2013).

### Administration Routes

*In vivo* and clinical studies exploring the aforementioned therapeutic effects of curcumin have assessed various routes of administration (Ravindranath and Chandrasekhara, 1981; Garcea et al., 2005; Smith et al., 2010a; Smith et al., 2010b; Gou et al., 2011; Sun et al., 2011; Wang et al., 2012; Chowdhury et al., 2013; Dovigo et al., 2013; Phillips et al., 2013; Subhashini et al., 2013; Sun et al., 2013a). In most studies, curcumin was delivered by gavage, *per os* (PO), intra-peritoneal (IP), and intravenous (IV) administration, with IP and gavage delivery more often applied to animals rather than to humans (**Table 3**) (Ravindranath and Chandrasekhara, 1980; Shoba et al., 1998; Pan et al., 1999; Perkins et al., 2002; Sharma et al., 2004; Garcea et al., 2005; Lao et al., 2006; Maiti et al., 2007; Marczylo et al., 2007; Yang et al., 2007; Sun et al., 2013a). Nonetheless, various other administration routes have been investigated, including sub-cutaneous (SC), topical, and nasal delivery (Shahani et al., 2010; Wang et al., 2012; Dovigo et al., 2013; Subhashini et al., 2013; Sun et al., 2013b).

**Table 3:** Concentration of unformulated curcumin in plasma, serum, and various tissues of rats and humans after different routes of administration (PO, IP, and IV).

Species	Route	Dose	Concentration of unformulated curcumin in the plasma, serum and various tissues			Reference
			**Plasma**			
Rat	PO	1000 mg/kg	15 ng/mL			(Prasad et al., 2014)
		500 mg/kg	60 ng/mL			(Yang et al., 2007)
		100mg/kg	220 ng/mL			(Pan et al., 1999)
	IP	100mg/kg	2250 ng/mL			(Pan et al., 1999)
		100mg/kg	2 nmol/mL			(Perkins et al., 2002)
	IV	10 mg/kg	360 ng/mL			(Yang et al., 2007)
		2 mg/kg	6600 ng/mL			(Sun et al., 2013a)
Human	PO	4000-8000 mg	410-1750 nM			(Cheng et al., 2001)
		3600 mg	11.1 ng/mL			(Sharma et al., 2004)
			**Serum**			
Rat	PO	2000mg/kg	1350 ng/mL			(Shoba et al., 1998)
		1000mg/kg	500 ng/mL			(Maiti et al., 2007)
		340 mg/kg	6.5 nM			(Marczylo et al., 2007)
Human		12000 mg	51.2 ng/mL			(Lao et al., 2006)
		10000 mg	50.5 ng/mL			(Lao et al., 2006)
		4000-8000 mg	400-3600 nM			(Cheng et al., 2001)
		2000 mg/kg	6 ng/mL			(Shoba et al., 1998)
		400-3600 mg	7-20 nmol/g			(Garcea et al., 2005)
			**Gastrointestinal (GI) tract**			
Rat	PO	2000mg/kg	**Averaged GI tract concentration**			
			42125 ng/g	Small intestine	58600 ng/g	(Ravindranath and Chandrasekhara, 1980)
				Stomach	53300 ng/g	(Ravindranath and Chandrasekhara, 1980)
				Cecum	51500 ng/g	(Ravindranath and Chandrasekhara, 1980)
				Large intestine	5100 ng/g	(Ravindranath and Chandrasekhara, 1980)
			**Intestine**			
	IP	100mg/kg	117040 ng/g			(Pan et al., 1999)
			**Intestinal mucosa**			
	IP	100mg/kg	200 nmol/g			(Perkins et al., 2002)
			**Other organs and tissues**			
Rat	IP	100mg/kg	**Liver**			
			26900 ng/g			(Pan et al., 1999)
			73 nmol/g			(Perkins et al., 2002)
			**Spleen**			
			26010 ng/g			(Pan et al., 1999)
			**Kidney**			
			7510 ng/g			(Pan et al., 1999)
			78 nmol/g			(Perkins et al., 2002)
			**Lungs**			
			16 nmol/g			(Perkins et al., 2002)
			**Heart**			
			9.1 nmol/g			(Perkins et al., 2002)
			**Muscle**			
			8.4 nmol/g			(Perkins et al., 2002)
			**Brain**			
			400 ng/g			(Pan et al., 1999)
			2.9 nmol/g			(Perkins et al., 2002)

### Curcumin’s Pharmacokinetics

Despite curcumin’s pleiotropic health attributes and proven safety, *in vivo* efficacy is hindered by its poor pharmacokinetic (PK) properties, mainly due to its low bioavailability (Anand et al., 2007; Prasad et al., 2014). The latter is due to numerous factors, including its low free serum concentrations, limited tissue distribution, short half-life, and apparent rapid metabolism and elimination (Karlowicz-Bodalska et al., 2017). Most curcumin is rapidly metabolized (*via* glucuronidation and sulfation) in the liver and intestine, leaving a small quantity detectable in tissues (Anand et al., 2007; Prasad et al., 2014). The major route of elimination of curcumin after PO administration is feces (Anand et al., 2007). The urinary excretion of curcumin or of its metabolites (glucuronide and sulfate derivatives) is very low regardless of the oral dose (Anand et al., 2007). Biliary excretion of curcumin was only seen in rats after IV and IP administration (Anand et al., 2007).

### Curcumin Bioavailability: From In Vivo Findings to Clinical Applications

The *in vivo* efficacy of any therapeutic compound is determined by the bioavailability of its free (unbound) concentration, not only in blood but also surrounding the therapeutic target (Smith et al., 2010a). Numerous studies confirmed negligible amounts of unformulated curcumin in the serum and plasma of rats and humans after gavage, PO, IP, and IV administration (Ravindranath and Chandrasekhara, 1980; Ravindranath and Chandrasekhara, 1981; Shoba et al., 1998; Pan et al., 1999; Cheng et al., 2001; Perkins et al., 2002; Sharma et al., 2004; Garcea et al., 2005; Lao et al., 2006; Maiti et al., 2007; Marczylo et al., 2007; Yang et al., 2007; Chang et al., 2013; Sun et al., 2013a). Comparison of the data generated from these studies indicates that serum/plasma curcumin levels in rodents and humans do not necessarily correlate (**Table 3**). The available data also emphasizes the role of the administration route on achievable serum levels. For example, plasma concentrations of 60 and 360 ng/mL were achieved, respectively, in rats following unformulated curcumin administration (500 mg/kg, PO and 10 mg/kg, IV, respectively) (Yang et al., 2007). Thus, it seems that greater serum/plasma concentrations of unformulated curcumin are achieved *via* IV and IP administration, better than PO and gavage administration (Anand et al., 2007) (**Table 3**). Similar observations were found on tissue distribution and administration route. For example, IP administration of low doses of unformulated curcumin (100 mg/kg) gave a much greater distribution of the compound in the intestines (~2.8 increase) when compared with much higher doses administered PO (2000 mg/kg) (Ravindranath and Chandrasekhara, 1981; Pan et al., 1999) (**Table 3**). Moreover, IP administration of unformulated curcumin inhibited the pro-fibrotic effects (inflammation and collagen deposition) and reduced the idiopathic pulmonary fibrosis progression, while PO administration was revealed to be ineffective (Smith et al., 2010b). This highlights the need for selecting a proper administration route for the same curcumin formulation to attain the therapeutic target and achieve proper *in vivo* efficacy. In addition, studies in rats have shown a dose-dependent limitation to the bioavailability of unformulated curcumin for the same route of administration, where increasing the administered dose has not resulted in an increase in tissue concentrations (Ravindranath and Chandrasekhara, 1981). The distribution of unformulated curcumin was also variable among the different tissues. The highest amounts of unformulated curcumin were identified in the gut, stomach, liver, and spleen of rats (Ravindranath and Chandrasekhara, 1981; Pan et al., 1999; Perkins et al., 2002) (**Table 3**). The amounts of unformulated curcumin found in the liver and spleen were lower than those identified in the stomach and intestine by a factor of ~3 to 5 (Pan et al., 1999; Perkins et al., 2002) (**Table 3**). In the gastrointestinal (GI) tract of mice, the highest amount of unformulated curcumin was identified in the small intestines (Ravindranath and Chandrasekhara, 1980). Additionally, the kidney, heart, lungs, and muscles showed moderate amounts of unformulated curcumin (in descending order), while trace curcumin amounts were identified in the brain (Pan et al., 1999; Perkins et al., 2002) (**Table 3**).

### Curcumin Features for Industrial Formulation

Commercial curcumin contains approximately 77% diferuloylmethane, 17% demethoxycurcumin, and 6% bisdemethoxycurcumin (Anand et al., 2007). Based on the Lipinsky rule of five, it seems that the molecular weight (MW) of curcumin allows for its GI absorption (MW of 368.38 Da < 500 Da) (Banks, 2009). However, numerous studies have reported that curcumin is poorly absorbed from the GI tract (Suresh and Srinivasan, 2010; Karlowicz-Bodalska et al., 2017). Indeed, as curcumin is a lipophilic compound (Karlowicz-Bodalska et al., 2017), its lipophilicity plays a key role in its absorption, distribution, metabolism, and elimination (ADME). Nonetheless, its lipophilicity favors its uptake by the peripheral tissues, which in turn lowers the free curcumin concentrations in the blood (Pan et al., 1999; Cheng et al., 2001; Sharma et al., 2004; Yang et al., 2007; Banks, 2009; Chang et al., 2013; Sun et al., 2013a; Karlowicz-Bodalska et al., 2017). For instance, its lipophilicity and low molecular weight makes curcumin a good substrate for the P-glycoprotein that effluxes substances from the blood–brain barrier (BBB) (Banks, 2009). The P-glycoprotein can greatly limit the rate of substances uptake, such as curcumin, by the BBB, which is a major obstacle in drug development (Banks, 2009). This explains the trace curcumin amounts found in the brain (Pan et al., 1999; Perkins et al., 2002). Thus, in order to achieve a proper *in vivo* efficacy, it is important to attain the PK/PD targets. The amounts of free curcumin detected in the blood or in the target tissues should be enhanced using new formulations depending on the required molecular target. In addition to new formulations, a proper selection of the administration route is crucial to enhance the curcumin bioavailability. Different curcumin formulations have been designed to enhance its bioavailability, including synthetic curcuminoids, nanoparticles, liposomes, micelles, and phospholipid complexes. These new curcumin formulations not only enhance its bioavailability but also allow for longer circulation, better permeability, and resistance to metabolic processes (Anand et al., 2007; Suresh and Srinivasan, 2010; Prasad et al., 2014). In addition, the effect of these new formulations on curcumin bio-efficacy has also been reviewed in the literature (Anand et al., 2007; Prasad et al., 2014), where, among others, the co-administration of piperine as an adjuvant with curcumin enhances its bioavailability (Anand et al., 2007; Suresh and Srinivasan, 2010; Prasad et al., 2014).

### Curcumin Safety and Quality Parameters

Numerous reports have indicated that curcumin exhibits multiple pharmacological activities, such as antioxidant and antimicrobial properties. With a long-established safety record, curcumin has been found to be quite safe in animals and humans, even at doses up to 8 g/day. Consequently, this substance was declared as GRAS by the FDA.

Studies on curcumin toxicity have been conducted *in vitro, in vivo*, and in humans, where, despite its well-established safety, some reports have highlighted deleterious side effects under certain conditions, as briefly discussed below.

#### In Vitro Data

*In vitro* experiments have demonstrated potential adverse effects. Sakano and Kawanishi (2002) demonstrated that curcumin, in the presence of copper and cytochrome p450 isoenzymes, leads to DNA fragmentation and base damage. In addition, Frank et al. (2003) proved that, in a rat model of liver cancer, curcumin bound to copper and did not inhibit spontaneous hepatic tumor formation. The enhanced toxicity and oxidative stress may be explained by the excess load of copper.

#### Acute Toxicity

No acute toxicity was described in animals. Vareed et al. (2008) evaluated the pharmacokinetic parameters of a curcumin preparation in healthy human volunteers after a single oral dose. Some side effects were reported after curcumin administration (at 10 and 12 g). These side effects were qualified as non-serious, as they correspond to grade 1 of the WHO classification of toxicity grades. According to this study, curcumin is considered as safe to use.

Cheng et al. (2001) tested several curcumin doses (500 mg, 1, 2, 4, 8, and 12 g/day) in a phase I clinical trial with 25 patients presenting with a high risk of cancer or pre-malignant lesions. Curcumin was taken orally for three months. Cheng et al. (2001) found no toxic effects even at doses of 8 g curcumin/day. However, doses higher than 8 g/day was found to be intolerable by the treated patients.

#### Chronic Toxicity

##### In Animals

Adding turmeric (0.5%) or curcumin (0.015%) to mice diet did not cause chromosomal aberrations and did not significantly affect pregnancy rates, the amount of alive and dead embryos, total implants, nor mutagenic index (Vijayalaxmi, 1980). Furthermore, according to Ammon and Wahl (1991), high curcumin doses (100 mg/kg P.V.) produce ulcerogenic effect in rats.

The National Toxicology Program (1993) assessed the short- and long-term toxicity of an organic extract from turmeric, called turmeric oleoresin. Rats and mice were fed diets containing turmeric extract at varying doses (1000, 5000, 10,000, 25,000, or 50,000 mg/mL equivalent to daily doses of 50, 250, 480, 1300, or 2600 mg/kg b.w) for two periods: 13 weeks or 2 years. No mortality was noted in either male and female rats, neither in the 13-week study nor in the 2-year study. It turned out that in the 13-week study, turmeric oleoresin induced a relative increase in liver weight, stained fur, discolored faces, and hyperplasia of mucosal epithelium in the cecum and colon of rats receiving the higher concentration of turmeric extract. The turmeric oleoresin administration also did not cause carcinogenic lesions. In the 2-year study, rats and mice fed with 50,000 mg/mL turmeric oleoresin developed ulcers, chronic active inflammation, cecum hyperplasia, and forestomach, increased incidences of clitoral gland adenomas, and developed hepatocellular and intestinal carcinoma (National Toxicology Program, 1993).

In another study developed in mice, the dietary administration of the whole spice turmeric (0.2%, 1.0%, 5.0%) or ethanolic turmeric extract (0.05%, 0.25%) for 14 days caused hepatotoxicity (Kandarkar et al., 1998). In a study, Deshpande et al. (1998) gave Wistar rats and female Swiss mice turmeric (0, 1 and 5%) and ethanolic turmeric extract (0, 0.05 and 0.25%) through their diet for 14 and/or 90 days. Hepatotoxicity, body weight gain reduction, and alterations in absolute and/or relative liver weights were detected in mice and rats fed with a high dose of turmeric for longer periods. Lower turmeric doses (i.e. 0.2 or 1%) for 14 days also exhibited hepatotoxicity in mice, being more vulnerable to turmeric-induced hepatotoxicity than rats (Deshpande et al., 1998). In preclinical systematic safety studies commissioned by the National Cancer Institute (NCI), no toxic effects were stated at doses of 3.5 g/kg given for 3 months in rats, dogs, and monkeys (Sharma et al., 2007).

##### In Humans

Sharma et al. (2007) noted that 1.5 g of turmeric powder per day (about 150 mg of curcumin, average consumption in India) did not exhibit any side effects in humans. In addition, Lal et al. (2000) reported that 1.125 mg of curcumin/day did not show any side effects in humans.

A study assessing the curcumin pharmacological aspects reported that it is distinguished by its safety of use and has an effect on the treatment of ulcerative colitis (Aggarwal and Sung, 2009).

According to Chainani-Wu (2003), who assessed curcumin’s safety and anti-inflammatory activity over the years 1966 to 2002, in a phase 1 human trial with 25 subjects using up to 8,000 mg of curcumin/day for 3 months, revealed no toxic effects. Moreover, pilot phase I safety trials concluded that curcumin is safe when consumed at a daily dose of 12 g for 3 months (Goel et al., 2008).

#### Side Effects, Interactions, Contra-Indications, and Precautions

##### Dermatitis and Allergies

Swierczynska and Krecisz (1998) have identified allergic contact dermatitis and urticaria especially following direct curcumin exposure to the skin or scalp. Liddle et al. (2006) reported a few cases of allergic contact dermatitis and two cases of contact urticaria. These cases were classified into immunologic (immunoglobulinE mediated) and nonimmunologic with the presence of inflammatory mediators (histamine, prostaglandins, leukotrienes). The immunological forms extend beyond the contact zone, whereas nonimmunologic forms remain limited to the area of contact and can evolve into chronic dermatitis (Liddle et al., 2006).

People already suffering from allergies to plants of the *Curcuma* genus are more likely than others to have an allergic reaction to turmeric (spice) or any of its constituents.

##### Carcinogenesis

Dance-Barnes et al. (2009) revealed that curcumin may enhance ROS formation and promote lung cancer in mice. Higher curcumin doses increase ROS cell levels, playing an important role in carcinogenesis (Ahsan and Hadi, 1998; Fang et al., 2005; Lopez-Lazaro, 2008).

##### Reproduction

It has been reported that curcumin may alter fertility by inhibiting human sperm motility and has a potential application as a novel intravaginal contraceptive (Rithaporn et al., 2003). According to Garg (1974), the oral administration of petroleum ether and aqueous extracts from turmeric rhizomes had a 100% anti-fertility activity in rats. Another study highlighted the ability to inhibit implantation by these extracts (Garg et al., 1978). In addition, it has been shown that curcumin suppressed the growth of hamster flank organs by inhibiting 5a-reductase, which converts testosterone into 5a-dihydrotestosterone (Liao et al., 2001).

##### Digestive Disorders

In high doses, turmeric is likely to cause GI disorders, such as abdominal pain, nausea, and diarrhea, that can be minimized through curcumin consumption at mealtimes. Nausea, diarrhea, and an increase in serum alkaline phosphatase and lactate dehydrogenase contents were experienced in patients receiving 0.45 to 3.6 g/day curcumin for 1 to 4 months (Sharma et al., 2004). In another study, daily curcumin intake of 8 g for 2 months in patients with advanced pancreatic cancers led to abdominal pain as the predominant side effect. Digestive ulcerations were also reported (Aggarwal and Sung, 2009). Diarrhea, headache, rash, and yellow stool were also noted in seven subjects receiving escalating doses (from 500 to 12,000 mg) of curcumin (Lao et al., 2006).

##### Synergistic Effects

Turmeric, through its major active constituent, curcumin, has synergistic effects, and can potentiate the effect of other drugs/substances. In fact, synergistic effects have been found when curcumin is combined with antibiotics (norfloxacin) (Pavithra et al., 2009), anti-inflammatories (Nandal et al., 2009), with certain cytotoxic drugs, with chemotherapy (Aggarwal et al., 2005; Kamat et al., 2007; Kunnumakkara et al., 2007; Lin et al., 2007), or when diet supplies contain other polyphenol derivatives (Strimpakos and Sharma, 2008). When administrated with paclitaxel (Taxol), curcumin significantly inhibited breast and lung cancer metastasis to a higher degree than curcumin or paclitaxel used alone (Aggarwal et al., 2005).

##### Inhibitory Effects

Although several studies have shown that curcumin raises the effect of chemotherapeutic agents, sometimes it might antagonize and inhibit their antitumor efficacy (Lopez-Lazaro, 2008). For example, curcumin combined with cyclophosphamide annulled the effect of cyclophosphamide and then inhibited the tumor size reduction in mice (Somasundaram et al., 2002). It is not clear why curcumin exhibits such contrasting activities (Sandur et al., 2007a; Sandur et al., 2007b), but it has been proposed that this may be related to curcumin concentration that plays a role in switching from its antioxidant to prooxidant effect. Curcumin may also interfere with irinotecan absorption and efficacy (Johnson and Mukhtar, 2007).

##### Interactions

Turmeric’s beneficial effects are undeniable; however, its consumption may interact with certain drugs and lead to several risks. For example, turmeric exerts an anticoagulant activity and inhibitory effects on platelet aggregation due to its antithrombotic properties; therefore, it is essential to consider this effect, given the fact that it potentiates the action of antiplatelet drugs (Shah et al., 1999)

Coupled with Ginkgo biloba or garlic or with an anticoagulant, such as aspirin (acetylsalicylic acid), clopidogrel (Plavix), dipyridamole (Persantine), ticlopidine (Ticlid), warfarin (Coumadine), or enoxaparin (Lovenox), turmeric can intensify its action, leading to serious consequences, such as hemorrhages. Moreover, according to animal studies, turmeric can lower the blood sugar and, as a result, have additive effects with oral antidiabetic drugs and insulin. Also, as turmeric can decrease blood pressure, it has additive effects if taken with antihypertensives. Lastly, turmeric, known for its digestive properties, increases stomach acid levels. However, when associated with antacids, such as cimetidine (Tagamet), famotidine (Pepcid), ranitidine (Zantac) and omeprazole, it can inhibit their effectiveness.

##### Contra-Indications

Turmeric use is not recommended for individuals allergic to Zingiberaceae plants. Regarding pregnancy and breastfeeding, turmeric has historically been considered safe when used as a spice. However, it has already been shown to cause uterine stimulation and thus may stimulate menstruation onset. Despite the fact that curcumin intake does not affect fetal development, turmeric use is not recommended during pregnancy and breastfeeding, and precautions should be taken due to the lack of clinical studies. In addition, curcumin may stimulate gallbladder contractions and cause gallstones development. Despite the lack of human studies, curcumin use is not recommended in patients with gallstones or biliary obstruction (Rasyid and Lelo, 1999).

In short, turmeric and curcumin appear to be extremely safe and well-tolerated, even at high doses (up to 8 g), without toxic effects. Moreover, epidemiological data have shown a low incidence of several types of cancer in individuals who regularly consume curcumin (Aggarwal and Sung, 2009). However, the safety of curcumin should be further explored, and long-term studies are needed for a better evaluation of possible adverse effects and to fully determine its toxic potential.

##### Precautions

Given that curcumin can inhibit the antitumor activity of chemotherapeutics, its use during chemotherapy should only be done under medical supervision. Similarly, vigilance is a must in patients allergic to turmeric or to any of its constituents; similar attention should be paid to patients with blood coagulation disorders or under anticoagulants treatment. In these cases, dose adjustments are needed, while in cases of a scheduled surgery, curcumin use should be stopped. Finally, turmeric should be applied with caution in individuals with diabetes or hypoglycemia or when treated with drugs that lower blood glucose levels.

## Curcumin Multidimensional Applications: Current Trends and Future Demands

### Food Attractiveness Optimization

Food dyes have been used since ancient times and are currently a trending topic in the food industry. Besides rendering foodstuffs more appealing and delightful, they affect shelf-life and microbiological quality and security. Following food industry expansion, many synthetic colorants were developed to ameliorate foodstuff features, but, over time, most of them were disqualified due to short/long term side effects, toxicity, and potential carcinogenic effects and other health injuries (Carocho et al., 2014; Amchova et al., 2015). Therefore, together with the increasing consumers’ demand for more delightful products, natural food colorants have emerged as a greater option. As a matter of fact, natural pigments, as well as synthetic analogues, are effective, but may also be safe. In addition, they are able to induce both health benefits and functional and/or additional properties, including preservative effects (Martins et al., 2016). Modern consumers are also looking for novel “functional foods” (Bagchi, 2006), with remarkable efficacy in preventing the onset of certain diseases. These are often chosen based on their abilities to prevent disease, more so than for their ability to reduce the risk of disease or to enhance organoleptic properties (Siro et al., 2008). Also, the so-termed “functional ingredients”, such as food pigments or colorants and other natural substances able to improve the nutritional status and acting as health promoters, are currently drawing consumers’ attention. Not surprisingly, curcumin is widely considered a good naturally-derived ingredient for functional foods formulation and helpful to prevent various chronic diseases (Xu et al., 2018).

In recent years, it has emerged that customers’ satisfaction is not only driven by taste, smell, appearance, and attractiveness but also by health effects, impact on quality of life, and ageing. Necessarily, the efforts of food industries are oriented towards the development of innovative strategies to meet both organoleptic and health demands. To achieve these purposes, modern industry applies a number of different techniques to ameliorate natural pigment extraction and to maintain their stability, in order to reduce the risk of color degradation and any subsequent loss of attractiveness. It has to be mentioned that efforts have also been directed to the development of differently designed packaging with the aim of improving the shelf-life of foodstuffs, thus limiting the use of additives, but also to develop safer and more appealing products for conscious consumers. However, direct collaboration with researchers and listening to consumer claims offers substantial help to food factories in pursuing these goals (Martins et al., 2016).

### Agro-Industrial Procedures to Offset Curcumin Instability and Low-Bioavailability

The raise in curcumin stability and bioavailability, together with the reduction of turmeric aroma, are currently the major issues pursued by scientific research to reach a wider use of curcumin both for food and pharmaceutical industries.

As a spice, turmeric extract possesses a characteristic pungent flavor predominantly due to *ar*-turmerone (2-methyl-6-(4-methylphenyl)-2-hepten-4-one), a constituent of turmeric oil; as well as curcumin, *ar*-turmerone is endowed with antimicrobial, antioxidant, and anticancer effects. Notably, the turmeric aroma influences the particular taste of the food product to which is added, consequently affecting the overall foodstuff’s sensory desirability and so representing a con for the use of turmeric extract as a food additive (Laokuldilok et al., 2016).

Curcumin is susceptible to light, unstable at pH>7, and, as with many other natural antioxidant substances, undergoes oxidative degradation (Nelson et al., 2017). In addition, it is insoluble in water and soluble in organic solvents, thereby limiting bioavailability in human aqueous body fluids. Taken together, these shortcomings complicate and limit the potential application of turmeric and curcumin for different uses; so, different approaches have been applied to overcome these drawbacks.

Encapsulation is a generally used technique for food colorants/additives, since it provides the means to convert liquids into solids, alter colloidal and surface properties, offer environmental protection, and control the released features or the coated materials’ availability (Özkan and Bilek, 2014). A number of studies dealing with turmeric’s microencapsulation have been published, differing on the materials used and production method. To name a few, Cano‐Higuita et al., 2015 proposed a study to explore the modifications induced: (a) by different formulations of wall materials, such as binary or ternary mixtures constituted by gum Arabic, maltodextrin, and modified starch and (b) by different drying methods in the stability of microcapsules containing turmeric oleoresin. The authors found that the ternary blend was more effective to prevent curcumin loss and color changes in the microcapsules compared to the binary mixture. As regards the drying method, curcumin retention during lyophilization was better than spray drying, but contrary behavior was found during storage; conversely, spray‐dried curcumin microcapsules show higher retention after 8 weeks under incident light (Cano-Higuita et al., 2015).

Laokuldilok et al. (2016) investigated the masking properties of turmeric combined with a binary mixture of wall material, i.e. brown rice flour (BRF) and beta-cyclodextrin (b-CD). The materials were accurately chosen; BRF exerts health benefits beyond basic nutrition to human health, while b-CD possess an effective masking ability. The study showed that microcapsules consisting of 5% of core loading (based on 7% of gelatinized BRF solution) added to 20 g/L of b-CD as optimal formulation, were able to produce powder of high curcuminoids encapsulation with low volatile release, moisture content, and hygroscopicity. Thus, this novel encapsulation blend of carrier agents has been proven to possess a high aroma masking property together with a high retention of bioactive compounds (Laokuldilok et al., 2016).

The extremely low hydrolytic degradation rate of curcumin encapsulated inside polymeric particles suggests that encapsulation, particularly nanoencapsulation (particles <100 nm) (Murthy et al., 2018), is a valid method to ameliorate the high grade of curcumin instability and poor bioavailability.

As already mentioned, the major and urgent issue in curcumin consumption by itself is related to its poor bioavailability. This issue is due to an unfavorable pharmacokinetics (ADMET) profile characterized by low serum levels, limited tissue distribution, apparent rapid metabolism, and a short half-life (Anand et al., 2007; Sanidad et al., 2019). Extensive efforts were accomplished in the never-ending research of innovative and valid strategies to solve these limits.

One solution could be to enhance both solubility and dissolution rates (Hani and Shivakumar, 2014), reachable with cyclodextrin inclusion complexes, solid dispersions (SDs), and solid self-emulsifying drug-delivery systems (S-SEDDs). The increase in surface area by micronization, manipulating solid-state crystallinity, and prodrugs development are additional approaches to improve the aqueous solubility of curcumin (Ipar et al., 2019).

In recent years, nanotechnology-based drug delivery systems for curcumin, including liposomes, polymeric nanoparticles, microemulsions, and nanoemulsions, to cite a few, have been widely developed (Ipar et al., 2019; Sanidad et al., 2019). Nano-formulations possess a low *size/surface area* ratio, and offer some advantages, such as improved transport through the GI mucosa and the possibility of ensuring sustained and controlled release, as well as a targeted delivery. Another approach applied to improve curcumin’s pharmacokinetics is its co-administration with adjuvants that are able to narrow its metabolic pathways. The most known bioavailability enhancer for curcumin is piperine (bioperine); when associated with curcumin, piperine leads to an increase of 2000% in the bioavailability, both in animals and humans (Shoba et al., 1998; Han, 2011). However, this strategy has some limitations since piperine enhances the curcumin bioavailability through different mechanisms, such as inhibition of hepatic and intestinal glucuronidation and P-gp inhibition (Srinivasan, 2007; Han et al., 2008; Sri et al., 2019). Since glucuronidation is a process necessary to eliminate toxins and metabolized drugs, where P-gp is responsible for drug-efflux, a long-term use of piperine could lead to toxin accumulation and drug-toxicity with subsequent liver damage, especially in patients under pharmacological therapy (Burgos-Morón et al., 2010; Sri et al., 2019).

### Curcumin Health Concerns and Upcoming Strategies

Despite its many benefits on human health and its well-established safety profile, curcumin presents some health concerns. Several reports have proposed that curcumin may trigger toxicity under specific conditions. To give some examples, subjects treated with curcumin ranging from 0.45 to 12 g may suffer from nausea, diarrhea, headache, rush, yellow stool, and increased levels of both serum alkaline phosphatase and lactate dehydrogenase (Lao et al., 2006; Kocaadam and Şanlier, 2017).

In 2010, a work entitled “The dark side of curcumin” evidenced some doubts as regards the therapeutic potential of the so-called “golden spice”. The authors aimed to provide a review on curcumin’s negative properties in comparison to its beneficial effects (Burgos-Morón et al., 2010). For instance, some studies indicate a potential carcinogenic effect of curcumin at doses close to that evidencing beneficial effects, as it seems to induce DNA damage at both mitochondrial and nuclear levels. Carcinogenic activity has also been observed in mice fed with various doses of turmeric oleoresin for 3 months and 2 years. The mechanisms behind this effects seems to be related to the 2 α,β‐unsaturated ketones in the molecule’s chemical structure. These portions are known to establish covalent linkages with thiol groups of cysteine residues through a Michael addition reaction. This mechanism may lead to ROS production through modification of the antioxidant enzyme thioredoxin reductase. Moreover, it can induce topoisomerase II‐mediated DNA damage and can inactivate the “guardian of the genome p53”. In addition, curcumin was found to chelate iron, thus affecting its systemic metabolism, and to inhibit cytochrome P450, glutathione‐S‐transferase, and UDP‐glucuronosyltransferase activities. When inhibited, these three drug‐metabolizing enzymes may trigger toxicity due to the accumulation of xenobiotics (Burgos-Morón et al., 2010). Nevertheless, the main reasons leading to skeptical opinion on curcumin’s therapeutic benefits are its low bioavailability and instability, but also its potential interaction with key-proteins involved in metabolic pathways.

More recently, a detailed and clear-cut manuscript by Nelson et al. (2017) overviewed curcumin’s essential medicinal chemistry arguing that a reactive, unstable, nonbioavailable molecule could not be considered a poor prototypical lead compound for drug discovery. The work also evidenced that curcumin has recently been classified as both a pan assay interference compound (PAINS) and invalid metabolic panaceas (IMPS) candidate (Nelson et al., 2017).

Two critical scientific contributions were published in response to both manuscripts (Kurien et al., 2011; Bahadori and Demiray, 2017), with both highlighting that the limited number of cautionary reports that indicate research on curcumin as an “excess of the need” were numerically exceeded by the plethora of scientific manuscripts regularly published on this topic. Indeed, in 2015, a Curcumin Resource Database (CRDB) (Kumar et al., 2015) was set up, covering over 9000 publications, 500 patents, 1186 curcumin analogs, 195 molecular targets, and 176 varieties of *C. longa*, whose aim was to support the preclinical development of curcuminoids. Although these studies demonstrated curcumin’s therapeutic and protective potential in animals and humans, more extensive clinical studies are needed to clearly elucidate its human health effects (Xu et al., 2018).

## Concluding Remarks and Perspectives

Curcumin has a long history of use as a culinary spice and food dye, and even as an ingredient for multiple medicinal preparations in Ayurveda and Chinese medicine. Along the ages, progress in science has proven the wide spectrum of favorable effects curcumin has on human health. Nowadays, the “golden spice” is still used as a cooking ingredient, but modern technology allowed curcumin exploitation in many different applications related to food and health.

Over the past half-century, a high amount of distinct clinical trials have been accomplished to address curcumin’s efficacy, safety, and pharmacokinetics (Gupta et al., 2013; Subramani et al., 2018). Curcumin has been administered in several formulations, such as capsules, tablets, powder nanoparticles, liposomal encapsulation, and emulsions, with dose-escalating studies revealing that curcumin is safe at doses as high as 12 g/day for 3 months. Bioavailability has been the major curcumin therapeutic limitation, and to solve this problem new nanomedicine formulations have been developed to improve curcumin targeting, pharmacokinetics, efficacy, and cellular uptake (Salehi et al., 2020a; Salehi et al., 2020b). Its pleiotropic activities comprise a plethora of inflammatory diseases, such as cancer, CV disease, arthritis, atherosclerosis, diabetes, gastric disease, inflammatory bowel disease, psoriasis, acquired immunodeficiency syndrome, and so on. However, protective effects on hepatic conditions, chronic arsenic exposure, and alcohol intoxication have also been stated. At the same time, a wide range of molecular targets have been listed, such as pro-inflammatory cytokines, apoptotic proteins, NF-κB, COX-2, 5-LOX, STAT3, C-reactive protein, prostaglandin E(2), prostate-specific antigen, adhesion molecules, phosphorylase kinase, transforming growth factor-β, triglyceride, ET-1, creatinine, HO-1, AST, and ALT, which clearly justify its remarkable health effects. Interestingly, upcoming clinical applications are looking for curcumin-induced cognitive effects. Indeed, curcumin is known as a molecule able to prevent/weaken pathological processes leading to age‐related dementia, cognitive decline, or depression, despite conflicting conclusions that arouse some doubts regards its effectiveness. A recent paper reviewed the clinical trials on curcumin application for cognitive functions to assess its real efficacy (Zhu et al., 2018). Unfortunately, only six studies satisfied the inclusion criteria, so that data were insufficient to provide an accurate and precise estimation of curcumin outcomes in different individuals. However, results show that curcumin is safe and well tolerated and seems to be more useful in ameliorating cognitive function in elderly subjects than AD- and schizophrenia-related symptoms. In addition, different investigations have suggested curcumin to be a potential chemopreventive and anticancer treatment in human papilloma virus (HPV) infection, as well as in primary and malignant squamous cervical cancer (Teymouri et al., 2017). However, high-quality clinical trials are needed to unequivocally confirm the beneficial effects of curcumin in different clinical conditions. Going in that direction, the clinical trial registered with the number NCT02944578 is actually recruiting women with high grade squamous intraepithelial cervix lesion to validate topical application of curcumin in precancer cervical lesions (https://clinicaltrials.gov).

## Author Contributions

Conceptualization: JS-R, YT, MB, MM, BS, WCC, and NM. Validation investigation, resources, data curation, writing: all authors. Review and editing: WCC, JS-R, MM, AM, and NM. All authors contributed to the article and approved the submitted version.

## Conflict of Interest

WS and ND were employed by the company Aromatic Plant Research Center, Lehi, UT, United States.

The remaining authors declare that the research was conducted in the absence of any commercial or financial relationships that could be construed as a potential conflict of interest.
